# Anthropometric Measurements and Analysis for Objective Assessment of Gynecomastia Surgery Results

**DOI:** 10.1093/asjof/ojad073

**Published:** 2023-08-02

**Authors:** Karthik Ramasamy, Srivatsa M Shet, Pankaj Patil, Sanjib Tripathee, Nanthini Dhevi, Abisshek Raj Alagarasan

## Abstract

**Background:**

Gynecomastia surgery is one of the most common aesthetic procedures in males. There is a lack of objective analytical parameters to judge outcomes. In this study, the authors aim to introduce novel anthropometric measurements and analysis techniques for the objective assessment of surgical outcomes based on specific aesthetic targets.

**Objectives:**

To introduce quantification of gynecomastia surgery outcomes and compare the results among the different grades of gynecomastia.

**Methods:**

A total of 192 patients with gynecomastia were included. The patient cases were grouped according to grades and a set of anthropometric measurements were taken both before the operation and 6 months postoperatively. Liposuction and glandular excision were done through minimal incisions in all grades of gynecomastia, with the addition of ultrasound and nipple areola complex (NAC) lifting plaster in selected Grade 3 and all Grade 4 cases.

**Results:**

A statistically significant improvement in the perimeter of the triangular relationship of sternal notch and nipples, the elevation of the NAC, the reduction of the area of the NAC, and the correction of asymmetry of the chest were seen in all grades of gynecomastia, with increased differences in higher grades.

**Conclusions:**

A systematic objective analysis of the specific aesthetic targets helps to reliably compare results in a standard way and for carrying out improvisation of surgeons’ techniques. Meanwhile, this approach helps identifying the need for customization, eventually providing symmetric and aesthetically pleasing surgical results.

**Level of Evidence: 3:**

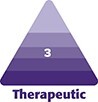

Gynecomastia is a very common condition in the general male population. Although it is largely a benign condition, it profoundly affects self-confidence, especially in young adults. The psychosocial effects of enlarged breasts in young adults and the distress they cause have been described in studies.^1^ Hence, it is the most commonly performed surgery among males in cosmetic surgery practice.^2^ Awareness about male chest aesthetics is growing among the public and surgeons irrespective of their age.^3-5^

To describe and quantify the results of gynecomastia surgery for specific aesthetic targets, the authors found a paucity of assessment tools. The existing tools do not quantify the elevation of the nipple areola complex (NAC) in a sagging gland, nor do they take into account how much asymmetry is corrected. In this study, the authors aim to introduce novel anthropometric measurements and analysis techniques for making an objective assessment of the postoperative results of gynecomastia surgery and to compare the postoperative results between different grades of gynecomastia.

## METHODS

The study was conducted at an aesthetic surgery center. The research proposal was submitted to the center's ethical board and approval was obtained. All primary gynecomastia surgeries performed between November 2021 and August 2022 were analyzed. Secondary surgeries and unilateral ones were excluded from the study. Written consent was provided, by which the patients agreed to the use and analysis of their data. A total of 192 patients were studied. The means of the demographic data are provided in Table 1.

**Table 1.** Comparison of Demographic Details Among Different Grades

Groups	Grade 1 (*n* = 17)		Grade 2 (*n* = 64)		Grade 3 (*n* = 82)		Grade 4 (*n* = 29)	
Parameter	Mean	SD	Mean	SD	Mean	SD	Mean	SD
Age	24.24	4.80	26.53	4.52	28.46	7.61	24.24	6.72
Weight	68.22	7.31	78.84	11.02	89.70	12.82	92.28	11.76
Height	1.72	0.06	1.73	0.06	1.73	0.07	1.74	0.06
BMI	23.04	3.00	26.19	3.27	29.94	3.24	30.60	3.82

SD, standard deviation.

These results are best achieved by defining specific aesthetic targets and appropriate surgical technique for each. The authors set the following aesthetic targets in all gynecomastia surgeries: (1) prominence of the pectoralis muscle region, (2) a flat NAC that sits at the level of the lower pectoralis muscle border (PMB), and (3) a reasonable symmetry of the chest concerning the position and size of NACs.

### Clinical Assessments and Planning of Customizations

After a routine clinical examination, patients were graded according to Rohrich classification^6^:

- Grade 1: Minimal hypertrophy without ptosis
- Grade 2: Moderate hypertrophy without ptosis
- Grade 3: Severe hypertrophy with Grade I ptosis
- Grade 4: Severe hypertrophy with Grade II or III ptosis

**Figure 1.**
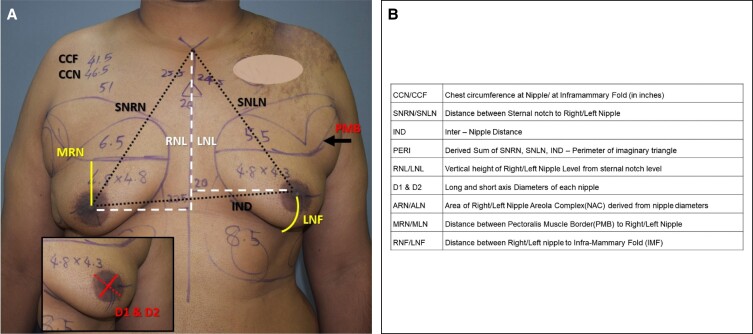
(A) A 25-year-old male patient with Grade 4 gynecomastia shown with preoperative markings and schematic markings. (B) Table showing abbreviations and descriptions of all markings and measurements.

A series of anthropometric measurements were noted, as shown in Figure 1. The PMB was marked to avoid/limit liposuction in that area. Focal abnormal fat deposits were marked. Two perpendicular diameters (D1 and D2) were noted to derive the area of the NAC. Chest circumference, distance from nipple to nipple, sternal notch to the nipple, and the vertical height between the sternal notch and the nipple were noted. The distance between the PMB and the nipple (MRN and MLN) was measured. In Grade 3 patients with an MRN/MLN distance >4.5 cm, ultrasound-assisted liposuction (UAL) was planned as done with all Grade 4 patients. Patient-specific asymmetries were noted by comparing right and left measurements. Quantitative differential use of ultrasound treatment was planned in case of an asymmetric sagging of the skin.

### Surgical Technique

Tumescent infiltration was done in a standard way through the axillary fold port. Fat removal was done using power-assisted liposuction. In select Grade 3 and all Grade 4 cases, UAL was used to facilitate better skin contraction and draping.^7^ The pectoral fascia was preserved to prevent the formation of skin adhesion to underlying tissue.^8^ A very thin disk of dermoglandular tissue was preserved under the NAC, which was not more than 2 to 3 mm thick.^9^ This was achieved by sharp dissection through a small inferolateral quadrant peri-areolar incision (1/4th circumference). The remaining part of the gland was extracted by the pull-through technique without skin excision.^10^ The wound was closed in layers after a thorough hemostasis. A pressure garment was applied immediately after surgery, and the patients were asked to continue using this garment for 6 weeks and advised to be worn 24/7 throughout this period. Sutures were removed after 1 week. To address skin laxity, the NAC lifting plaster was applied immediately after surgery and maintained for 1 to 2 weeks in all Grade 4 and selected Grade 3 cases.^11^ Postoperative measurements were taken again after 6 months.

### NAC Lifting Plaster Application Technique

NAC lifting plaster is a technique to treat skin redundancy in Grade 3 and Grade 4 gynecomastia.^11^ Lifting plasters are applied by lifting the NAC in 2 different vectors of pull, to be positioned on the pectoralis major muscle. The first plaster is applied obliquely on the chest above the NAC, with its vector of pull toward the suprasternal notch. The second plaster is applied vertically above the NAC with its vector of pull toward the clavicle, thus draping the redundant skin and the NAC to be positioned at the desired and aesthetically pleasing position in men.

## RESULTS

The patients in the study sample were aged between 13 and 49 years with a mean age of 26.8 years. The average follow-up time was 28.7 weeks with a range of 26 to 33 weeks. Preoperative and postoperative measurements were tabulated and compared. Paired *t* tests were done for obtaining the results of each parameter in each group (Table 2). Symmetry analysis was done with derived data from the nipple level, sternal notch to nipple distance, and the NAC area. The difference between right and left values was noted both preoperatively and postoperatively. The mean discrepancy was calculated and statistical analysis was done. The results are given in Table 3. There were 5 cases of patients with hematoma (2.6%), of which 3 needed open evacuation and 2 cases resolved after delayed needle aspiration. There were 3 cases of patients with seroma (1.56%), which resolved after 2 to 3 needle aspirations and compression. There were no other complications in our study such as NAC necrosis or infection.

**Table 2.** Comparison of Results of the Means and Paired *t*-Test for Each Parameter Among Different Grades

Groups		Grade 1 (*n* = 17)				Grade 2 (*n* = 64)				Grade 3 (*n* = 82)				Grade 4 (*n* = 29)			
Parameter	Unit	Mean	SD	Mean diff.	*P*-value	Mean	SD	Mean diff.	*P*-value	Mean	SD	Mean diff.	*P*-value	Mean	SD	Mean diff.	*P*-value
CCN	inch	35.62	1.40	0.76	<.001	39.05	2.75	1.96	<.001	41.83	3.02	2.59	<.001	43.07	3.59	3.02	<.001
CCNP	inch	34.85	1.57			37.09	2.29			39.24	2.79			40.05	3.28		
SNRN	cm	18.76	1.02	1.00	<.001	20.15	1.53	1.34	<.001	21.66	1.91	1.85	<.001	23.76	1.54	3.31	<.001
SNRNP	cm	17.76	0.90			18.81	1.33			19.81	1.56			20.45	1.55		
SNLN	cm	18.53	1.41	0.74	<.001	20.21	1.64	1.30	<.001	21.98	2.13	2.15	<.001	23.74	1.14	3.17	<.001
SNLNP	cm	17.79	1.19			18.91	1.32			19.82	1.56			20.57	1.55		
IND	cm	20.47	0.87	0.53	.003	21.74	1.70	1.23	<.001	22.76	2.20	1.99	<.001	24.03	2.00	2.16	<.001
INDP	cm	19.94	0.93			20.52	1.76			20.77	1.82			21.88	2.09		
Peri	cm	57.76	2.68	2.26	<.001	62.10	4.36	3.87	<.001	66.40	5.53	5.99	<.001	71.53	4.22	8.64	<.001
PeriP	cm	55.50	2.28			58.23	3.90			60.41	4.12			62.90	4.63		
RNL	cm	15.38	1.43	0.65	<.001	16.10	1.22	0.70	<.001	17.65	1.88	1.21	<.001	19.50	1.52	2.30	<.001
RNLP	cm	14.74	1.19			15.40	1.32			16.44	1.56			17.21	1.28		
LNL	cm	15.35	1.74	0.76	<.001	16.34	1.48	0.80	<.001	18.11	1.98	1.78	<.001	19.43	0.99	2.38	<.001
LNLP	cm	14.59	1.29			15.54	1.27			16.34	1.57			17.05	1.15		
ARN	cm^2^	7.15	3.00	1.82	<.001	8.61	3.24	2.75	<.001	10.41	2.62	3.91	<.001	13.06	4.73	6.41	<.001
ARNP	cm^2^	5.33	1.78			5.86	1.94			6.51	1.70			6.65	2.00		
ALN	cm^2^	7.15	2.84	1.71	<.001	8.58	3.49	2.80	<.001	10.88	2.76	4.21	<.001	11.76	4.18	4.99	<.001
ALNP	cm^2^	5.44	1.94			5.78	2.04			6.67	1.78			6.77	1.65		

All abbreviations with posttext “P” mean postsurgical measurements. ALN, area of left nipple; ARN, area of right nipple; CCN, chest circumference at nipple; IND, internipple distance; LNL, left nipple level; PERI, perimeter of the triangular relationship; RNL, right nipple level; SD, standard deviation; SNLN, sternal notch to left nipple; SNRN, sternal notch to right nipple.

**Table 3.** Comparison of the Effect of Surgery on the Preoperative and Postoperative Symmetry of the NAC Position as Well as Size Among Different Grades

Groups	Grade 1 (*n* = 17)			Grade 2 (*n* = 64)			Grade 3 (*n* = 82)			Grade 4 (*n* = 29)		
Parameter	Mean	SD	*P*-value	Mean	SD	*P*-value	Mean	SD	*P*-value	Mean	SD	*P*-value
AsSNN (cm)	0.41	0.64	.750	0.45	0.46	.094	0.58	0.45	.008	0.64	0.44	.008
AsSNNP (cm)	0.38	0.55		0.34	0.39		0.43	0.38		0.40	0.28	
AsNL (cm)	0.21	0.36	.496	0.55	0.57	.601	0.70	0.64	.003	0.83	0.70	.024
AsNLP (cm)	0.26	0.36		0.52	0.46		0.45	0.39		0.57	0.46	
AsAN (cm^2^)	0.36	0.32	.696	1.03	1.15	.001	1.29	1.28	<.001	1.85	1.87	.001
AsANP (cm^2^)	0.31	0.41		0.52	0.52		0.65	0.56		0.87	0.89	

All asymmetries calculated as a difference between right and left measurements. All subsequent abbreviations with added posttext “P” mean postsurgical measurements. AsSNN, asymmetry in sternal notch to nipple distance; AsNL, asymmetry in nipple levels; AsAN, asymmetry in area of nipples; NAC, nipple–areola complex.

## DISCUSSION

Multiple techniques are followed by plastic surgeons worldwide for correction of gynecomastia, such as a preference for the use of UAL, varying choices of incision, the thickness of the NAC flap retained, and fat grafting along the PMB. Whatever the technique, the final goal of the surgery is to obtain an aesthetic chest. However, a study with 448 patients has shown that a combination of all these techniques customized to the grade of gynecomastia provides superior patient satisfaction rates and reduction in postoperative complications.^8^

Improvisations in the surgical technique, and hence improved outcomes, are possible only by making a proper critical analysis of the results obtained. Outcome analysis of gynecomastia surgeries has been described so far with regard to volume reduction, patient satisfaction, rate of complications, and revision rates.^12^ With the advancement of techniques, good results for gland volume reduction are almost always achieved and complications are minor and transient without long-term effects. Apart from the comparison of chest circumference at the nipple level both pre and post surgery, not much attention has been paid to aesthetic outcome assessment in a systematic objective parameter–driven way. Such analysis is possible only after defining the above-mentioned aesthetic targets and by following assessment techniques that quantify the target results. Hence, we are describing 4 new assessment parameters to analyze the results of the gynecomastia correction surgery.

### The Perimeter of the Triangle Formed by Sternal Notch and Nipples for Improvement of Key Landmark Relations of Chest Aesthetics

**Figure 2.**
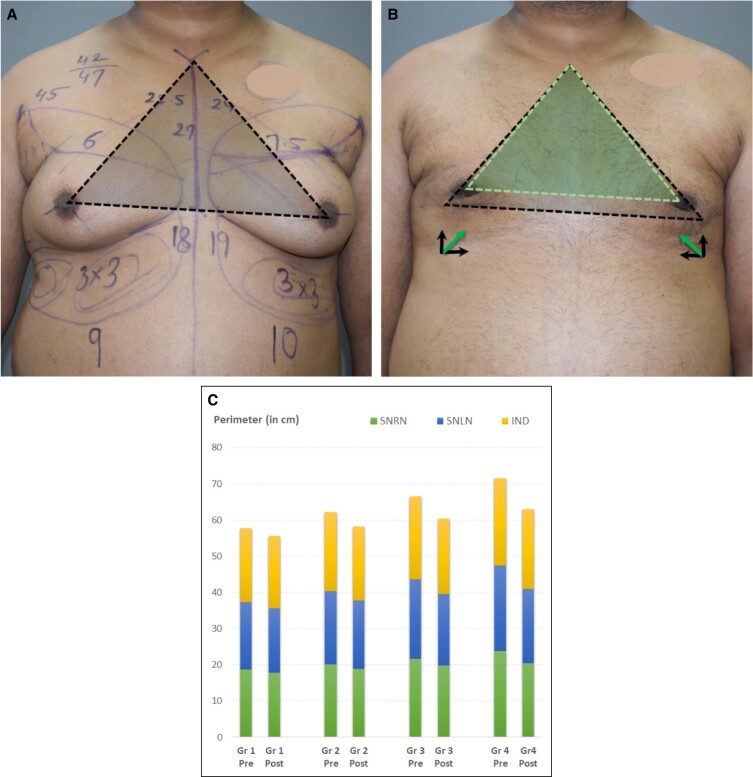
(A) Preoperative and (B) 6-month postoperative photographs of a 38-year-old male patient with Grade 3 gynecomastia. Note the schematic representation of the reduction in the perimeter of the triangle. Note the resultant vector of the movement of NACs (oblique arrow). (C) Chart showing a comparison of the results among different grades (Gr). IND, internipple distance; SNLN, sternal notch to left nipple; SNRN, sternal notch to right nipple.

The importance of subunit correction of aesthetics in the male chest has already been described in the literature.^2^ Contour correction is achieved by removing the gland, performing a liposuction of the lateral chest, preserving fat over the pectoralis muscle region, and/or fat grafting under the pectoralis muscle.^13^ This result is best represented by the conventional chest circumference improvement and by the perimeter of the triangle (PERI) formed by the key landmarks of the surgical area of interest, that is, the sternal notch, right nipple, and left nipple. This triangular relationship has been used previously by Penn for the assessment of aesthetics in females.^14^ Although the individual distance between these landmarks has been used in the literature for gynecomastia, a single measurement alone does not represent the overall result of the surgery. On the other hand, the perimeter takes into account both components of the NAC movement, that is, the medialization and the lift, as shown in Figure 2. The final vector of movement of the NAC is shown by the oblique arrow. A reduction in the perimeter can be seen and this quantifiably describes the result of the surgery. The improvement was statistically significant in all 4 grades (Table 2). The mean differences between pre- and postsurgical measurements were 2.26, 3.87, 5.99, and 8.64 cm for Grades 1, 2, 3, and 4, respectively. The difference in chest circumference at the nipple level also showed a similar trend of higher improvement in higher grades (Table 2).

### Right and Left Nipple Level From the Sternal Notch for Measurement of Correction of the Low Set NAC

Although the ideal location of the NAC is still debatable,^3^ there is only a limited opportunity to change its position through the UAL technique. Elevation of the NAC is one of the important targets of gynecomastia correction, especially in higher grades in which skin and NAC sagging are also higher. Adequate liposuction around the gland, use of energy devices, and manipulation of the skin excess by extensive undermining^9^ and NAC lifting plasters^11^ help in achieving elevation of the NAC. For assessment, previously sternal notch to nipple and acromioclavicular joint to nipple distances have been described in a study for a postoperative improvement assessment of nipple position in young Korean adults.^15^ Further for postoperative assessment, the midclavicular point to nipple distance has been described while studying the ideal NAC position in a normal male chest.^16^ This takes additional time and is prone for assessment errors and symmetry errors caused by asymmetric clavicle or shoulder positions. Absolute numbers in defining NAC positions^14,15,17^ do not do justice while reproducing them in populations of varied ethnicity, varied skeletal framework, and BMI. The correlation of NAC position with the PMB appears to have more rationale than numbers.

**Figure 3.**
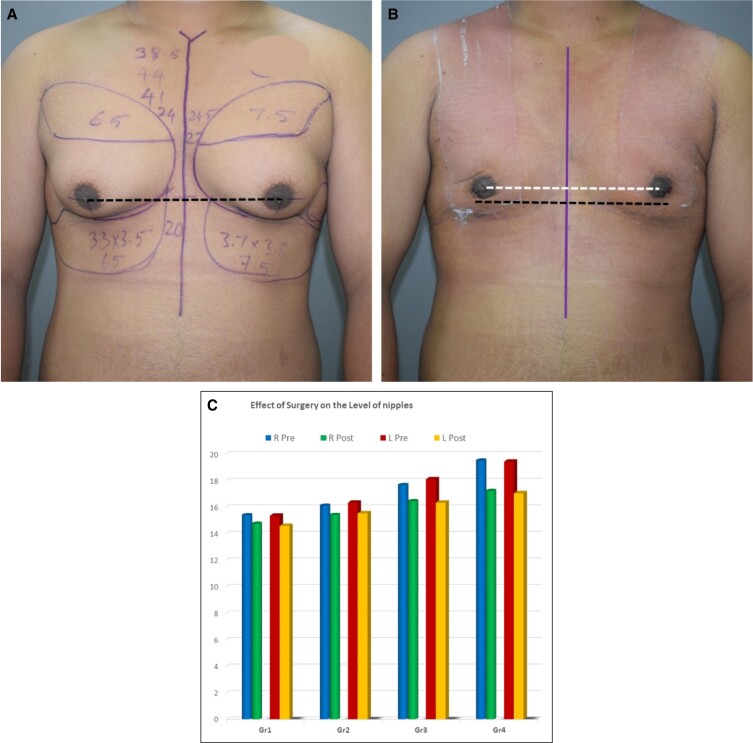
(A) Preoperative and (B) 2-week postoperative photographs of an 18-year-old male patient with Grade 2 Gynecomastia. The black line is the preoperative level of both nipples and the white line shows the postoperative level. (C) Chart showing a comparison of the net elevation of NACs as a result of surgery among different grades. Each grade (Gr) shows pre- and postoperative results of the right (R) and left (L) side.

Post surgery, the NAC moves superomedially (Figure 2B). But the visible result to the common eyes of the patient is the lift. According to the authors, the best way to assess this lift is by measuring the vertical distance between the level of the sternal notch and the level of the nipple (RNL and LNL). The amount of lift can be seen in Figure 3. The lift varies between the grades. It can be noted that the quantum of results increases as the grade becomes higher. The maximum improvements are seen in Grade 3 (1.21 cm right, 1.78 cm left) and Grade 4 gynecomastia (2.30 cm right, 2.38 cm left). This could be attributable to the undermining of the skin and the use of the UAL technique in all Grade 4 and selected Grade 3 patients having sagging of skin. The improvement was statistically significant in all 4 grades (Table 2).

### Area of Right and Left NAC for Measurement of Correction in the Size of the NAC

**Figure 4.**
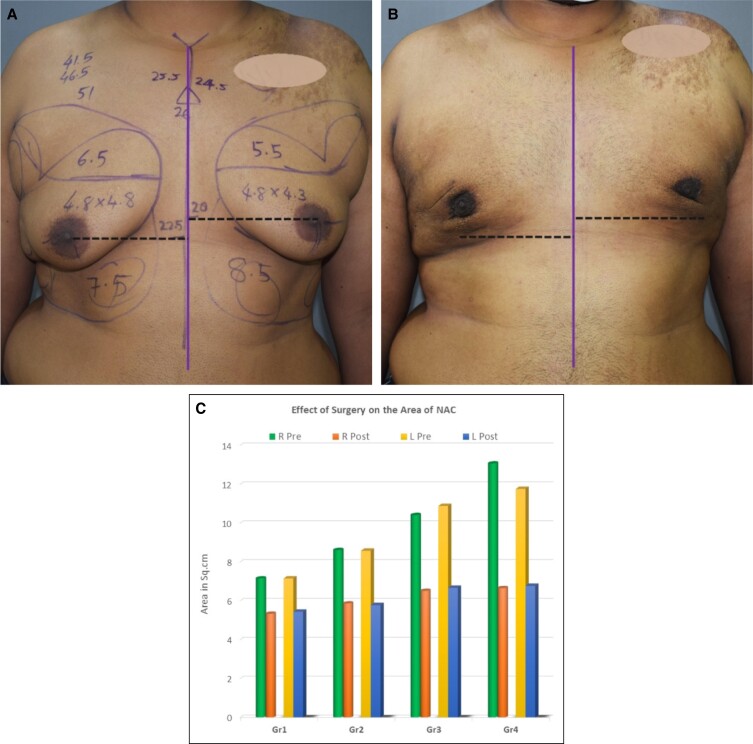
(A) Preoperative and (B) postoperative photographs of a 25-year-old male patient with Grade 4 gynecomastia. Note the change in the size of NACs. The black lines show the preoperative levels. This patient is also an example to show how shoulder drooping exaggerates asymmetry in the nipple levels. (C) Chart showing a comparison of a net reduction of the area of NACs as a result of surgery among different grades. Each grade (Gr) shows preoperative and postoperative results of right (R) and left (L) sides. NAC, nipple areola complex.

A large NAC is a feminine characteristic and its presence is obvious in gynecomastia, more so in Grade 3 and Grade 4 patients. This apparent NAC enlargement is contributed partly by the stretch of the large glandular element and partly by the actual growth of the skin. Thinning the NAC flap facilitates easy shrinking of the NAC during the healing process.^9^ The smaller the length of the incision, the more thinning of the NAC flap can be done safely without compromising the vascularity. Hence, we use an incision technique that is only 1/4th circumference of the NAC. In our study, none of the patients had any problems related to NAC vascularity. The mean differences in the pre- and postsurgical measurements of the area ranged from 1.71 to 6.41 cm^2^. An increasingly higher amount of area reduction was seen in higher grades of gynecomastia (Figure 4), and it was statistically significant in all grades (Table 2). This could be attributable partly to the elimination of forces exerted by the enlarged gland and partly to the fibrosis occurring during the healing phase of the thinned-out NAC flap.

### Correction of the Asymmetries of the Chest

Although asymmetry is the norm for the human body, be it the face or trunk, only a milder degree of asymmetry is acceptable. A gross asymmetry is noticeable and its correction should be a target in any aesthetic surgery. This can be identified and quantified during markings. The common asymmetries seen in gynecomastia are the vertical distance between the level of the sternal notch and the level of the nipple (AsSNN), asymmetry in the vertical height levels of the NAC (AsNL), asymmetry in the area of the NAC (AsAN), and asymmetries in the gland volume, pectoralis muscle volume, and fat volume. A customized approach to each patient is essential to achieve correction such as differential liposuction, use of UAL, and adjustment with lifting plasters.^11^ The authors’ approach toward customization is presented in Table 4.

**Table 4.** Authors’ Approach Toward the Quantification of Aesthetic and Anatomic Distortions and the Selection of the Custom Surgical Technique

Patient concerns	Element of aesthetic/anatomic distortion	Anthropometric measurements to quantify	Technique of correction followed by authors
Having suffered bullying from peers, low self-esteem, depression	The very presence of gynecomastia	Not available	Surgical correction +/− psychiatry consultation
Not able to wear appropriate fitting T shirts	Increased mammary gland volume and nipple projection	Difference between chest circumference at nipple and chest circumference at IMF	Liposuction and gland excision
	Fat deposits in the anterior axillary fold and lateral chest wall	Not available	Customized liposuction addressing fat pockets
Bouncing of gland and pain during jogging or fitness activities	Sagging gland (skin excess above and below NAC)	Muscle border to nipple distance (SNRN/SNLN, RNL/LNL, MRN/MLN)	Ultrasound-assisted liposuction in areas around the NAC and the use of NAC lifting plasters
Not able to go shirtless in the swimming pool or beach	Large-size areola	Area of NAC (ARN/ALN)	Thinning of the NAC flap
	Asymmetric size of the NAC	Difference in the right and left areas of the NAC (AsAN)	Custom differential use of UAL, skin undermining, thinning of the NAC flap, and NAC lifting plasters
	Asymmetric position of the NAC	Difference in right and left nipple levels from the sternal notch (AsSNN, AsNL)	
	Asymmetric size of the gland	Not available	

ALN, area of left nipple; ARN, area of right nipple; AsAN, asymmetry in the area of the NAC; AsNL, asymmetry in the vertical height levels of the NAC; AsSNN, vertical distance between the level of the sternal notch and the level of the nipple; IMF, inframammary fold; MLN, distance between the pectoralis muscle border and the left nipple; MRN, distance between the pectoralis muscle border and the right nipple; NAC, nipple areola complex; RNL/LNL, vertical distance between the level of the sternal notch and the level of the nipple; SNLN, sternal notch to left nipple; SNRN, sternal notch to right nipple; UAL, ultrasound-assisted liposuction.

**Figure 5.**
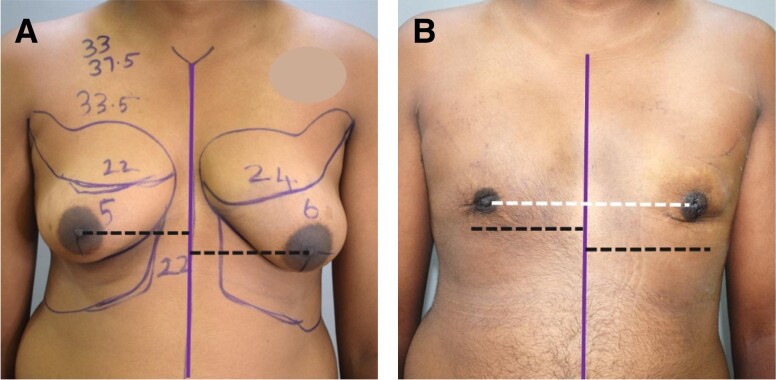
(A) Preoperative and (B) 6-month postoperative photographs of a 29-year-old male patient with Grade 4 gynecomastia. Note the correction of asymmetry concerning the vertical height levels of the NAC.

In our study, asymmetry was noted in all patients, with increasing asymmetry in higher grades. The postsurgical improvement was statistically significant for only Grade 3 and Grade 4 patients in our study (Table 3). There was a 0.64 cm^2^ mean area correction in the asymmetry of the NAC (AsAN) in Grade 3 patients, whereas it was 0.98 cm^2^ in Grade 4 patients. There was a 35.7% reduction in the discrepancy of nipple levels (AsNL) in Grade 3 patients and a 31.3% reduction in Grade 4 patients. An example of the importance of such correction is shown in Figure 5.

**Figure 6.**
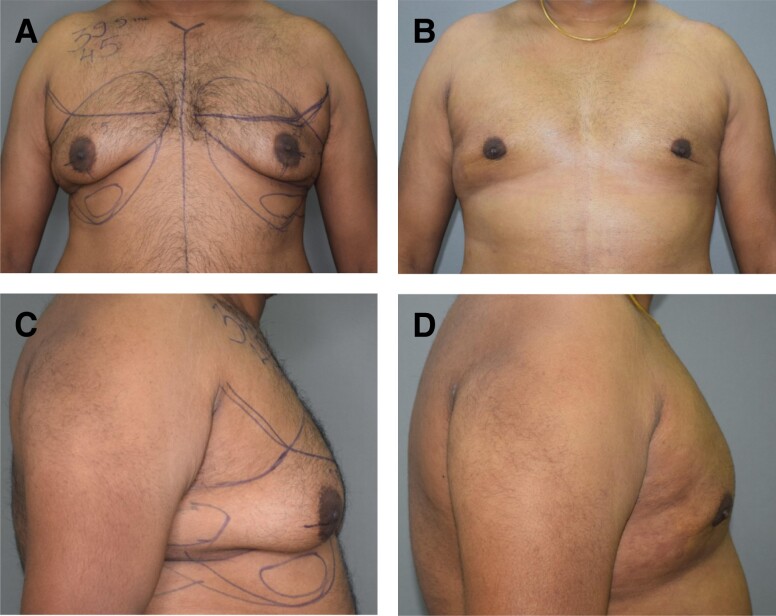
(A, C) Preoperative and (B, D) 7-month postoperative photographs of a 26-year-old male patient with Grade 4 gynecomastia. Note the elevation and supero-medialization of the NAC along with the reduction in size of the NAC. In the lateral view, the extent of correction in gland projection can be appreciated.

To summarize, attention to detail in the preoperative markings, systematic and customized intraoperative steps, and postoperative care can lead to good results in gynecomastia surgeries. One such result is shown in Figure 6. This study emphasizes symmetry as an aesthetic goal that can be achieved by a customized surgical approach. The new assessment parameters, namely perimeter, nipple levels, and the area of the NAC give a better picture of achievement of the said aesthetic targets of gynecomastia surgery. A critical analysis of these results from the aesthetic perspective will help surgeons improvise their techniques and to obtain better results in future surgeries. This will also help in counseling the patient and setting up an achievable expectation level in their mind. Finally, this will reflect on the overall patient satisfaction levels as well. The assessment tools and analysis techniques used in this study are simple, easily replicable, and suitable even in resource-limited settings.

### Limitations

The MRN/MLN measurement, the concept of customization based on the distance, and the arbitrary number 4.5 cm for the use of UAL are new concepts followed by the authors and hence they cannot be referenced. The authors have seen substantial improvement in the correction of sagging skin when compared with previous techniques on a clinical assessment basis. Further detailed comparative studies are required to prove the concept statistically. This study lacks the subjective outcome assessment (patient and surgeon reported) and its correlation with objective outcomes. Further studies are needed to explore this domain.

## CONCLUSIONS

The new objective assessment parameters, namely perimeter, nipple levels, and the area of NAC, provide a better outlook on the achievement of the aesthetic targets of gynecomastia surgery. A systematic objective analysis of the specific aesthetic targets helps in a reliable comparison of the results in a standard way and in improvisation of the surgeons’ techniques. Meanwhile, this approach helps identifying the need for customization, eventually providing symmetric and aesthetically pleasing surgical results. The assessment tools and analysis techniques used in this study are simple, easily replicable, and suitable even in resource-limited settings.
